# The Value of Local Heatwave Impact Assessment: A Case-Crossover Analysis of Hospital Emergency Department Presentations in Tasmania, Australia

**DOI:** 10.3390/ijerph16193715

**Published:** 2019-10-02

**Authors:** Sharon L. Campbell, Tomas A. Remenyi, Grant J. Williamson, Christopher J. White, Fay H. Johnston

**Affiliations:** 1 Menzies Institute for Medical Research, University of Tasmania, 1 Liverpool St, Hobart, Tasmania 7000, Australia; sharon.campbell@utas.edu.au; 2 Public Health Services, Department of Health (Tasmania), 25 Argyle St, Hobart, Tasmania 7000, Australia; 3 Antarctic Climate and Ecosystems Cooperative Research Centre, University of Tasmania, 20 Castray Esplanade, Hobart, Tasmania 7000, Australia; tom.remenyi@utas.edu.au (T.A.R.); chris.white@strath.ac.uk (C.J.W.); 4 School of Natural Sciences, University of Tasmania, Hobart, Tasmania 7001, Australia; grant.williamson@utas.edu.au; 5 Department of Civil and Environmental Engineering, University of Strathclyde, James Weir Building, 75 Montrose Street, Glasgow G1 1XJ, UK

**Keywords:** heatwave, extreme heat, morbidity, health effects, emergency presentation, case-crossover

## Abstract

Heatwaves have been identified as a threat to human health, with this impact projected to rise in a warming climate. Gaps in local knowledge can potentially undermine appropriate policy and preparedness actions. Using a case-crossover methodology, we examined the impact of heatwave events on hospital emergency department (ED) presentations in the two most populous regions of Tasmania, Australia, from 2008–2016. Using conditional logistic regression, we analyzed the relationship between ED presentations and severe/extreme heatwaves for the whole population, specific demographics including age, gender and socio-economic advantage, and diagnostic conditions that are known to be impacted in high temperatures. ED presentations increased by 5% (OR 1.05, 95% CI 1.01–1.09) across the whole population, by 13% (OR 1.13, 95% CI 1.03–1.24) for children 15 years and under, and by 19% (OR 1.19, 95% CI 1.04–1.36) for children 5 years and under. A less precise association in the same direction was found for those over 65 years. For diagnostic subgroups, non-significant increases in ED presentations were observed for asthma, diabetes, hypertension, and atrial fibrillation. These findings may assist ED surge capacity planning and public health preparedness and response activities for heatwave events in Tasmania, highlighting the importance of using local research to inform local practice.

## 1. Introduction

Anthropogenic climate change represents ‘an unacceptably high and potentially catastrophic risk to human health’ [1] (p. 1861). While climate change may not necessarily impact health through the introduction of new diseases or disorders, it is likely to expand and amplify existing health issues [2], presenting to the global population as a broad spectrum of health risks [3]. The Intergovernmental Panel on Climate Change describes global mean surface air temperature as rising over the last 100 years [4], which has led directly to an increase in frequency, intensity, and duration of extreme heat events since 1950 [5]. It is widely accepted that extreme heat, and specifically extreme heat events, have a detrimental effect on human health. In Australia, extreme heat is responsible for over 55% of total fatalities caused by natural events since 1900; more deaths than all other natural hazards combined [6]. 

Heatwaves have been studied across many parts of the world, although significant geographic gaps exist [7]. Heat-related illness and death does not present equally across populations, with some groups appearing more vulnerable than others [8]. Meta-analyses show that the greatest impacts appear likely for the elderly, children, and those with existing medical conditions, including cardiovascular diseases and mental illnesses [9,10].

Several methods exist to assess the extent to which extreme heat events impact human health; these include analyzing mortality data for the period of the event and shortly after [11]; analyzing morbidity indicators, such as ambulance dispatches, emergency hospital presentations, and hospital admissions [12]; or a combination of mortality and morbidity data [13,14]. Studies investigating the economic impact and work output have also emerged [15]. Studies of outcomes relating to heatwave-associated morbidity are, however, far less common than studies of mortality [7,8]. This is an important discrepancy, as mortality represents the extremes of health impacts, while understanding the association with other health outcomes is equally important for quantifying the greater impacts on the health care system and the society.

In Australia, several studies have examined the link between extreme heat and health outcomes [14,16,17,18,19,20,21], including for specific cohorts [22,23]. Across these studies, a positive association has been established between extreme heat events and increases in ambulance dispatches, hospital emergency department (ED) presentations, and deaths. These studies have principally concentrated on urban settings in the larger capital cities of Melbourne, Perth, Adelaide, Sydney, and Brisbane, which are all located in warmer climate regions. To date, no studies have been conducted specifically in the cooler climate regions of Australia, where health outcomes associated with heatwaves are unknown. 

### 1.1. Study Setting

Tasmania is an island state in Australia, located to the south of mainland Australia (40 °S–43 °S). The majority of the Tasmanian population reside in a regional or remote classified area [24]. The state’s total population in 2016 was 510,000, with most of the population residing in one of three major centers—Hobart, the capital, located in the southeast (population 204,000), Launceston in the north (population 84,000) or Burnie–Devonport in the northwest (population 70,000) [25]. There are slightly more females than males in Tasmania (98 males to 100 females), and the median age is 42.3 years, the highest of any Australian state or territory [25].

Tasmania has four major public hospitals, each with an emergency department, located in the most densely populated regions—one located in Hobart (Royal Hobart Hospital); one in Launceston (Launceston General Hospital); and two in the Burnie-Devonport region (the Mersey Community Hospital and the North West Regional Hospital).

Severe heatwaves are not a common feature of the Tasmanian summer experience, with average maximum summer temperatures of approximately 20 °C, some of the lowest found in Australia. However, Tasmania still experiences occasional extreme heat events. In late January 2009, for example, Tasmania experienced its hottest maximum temperature on record, reaching 42.2 °C at Scamander in the state’s northeast region. Several other towns in the north and northeast experienced similar maximum temperatures over the following days [26]. In 2013, Hobart experienced its hottest maximum temperature ever recorded (41.8 °C on 4 January) and several other highest summer temperature records were broken in the surrounding regions on that day [27]. This period in the southeast was also marked by severe wildfires [28].

When compared to other Australian jurisdictions, Tasmania has a greater proportion of people in higher risk groups identified as vulnerable to heat events. With 19.3% of the population over 65 years of age, Tasmania has the highest proportion of elderly residents [29], and the highest proportion of cardiovascular disease (7.7%), and long-term mental or behavioral problems (21%) [30]. Tasmania also has a higher proportion of people living in greatest disadvantage (33%) than any other Australian state and territory [30], with less than half of Tasmanian households having access to air-conditioning for cooling [31]. These factors potentially make the Tasmanian population more vulnerable to heatwaves when they do occur. 

As a compounding factor, typical Tasmanian weather patterns do not involve uniform increases and decreases in temperature throughout the spring-summer-autumn period. Due to its location within the westerly wind belt, and the consequent regular passage of cold frontal systems, Tasmanian meteorology is characterized by highly variable conditions and rapid shifts in temperature. For example, a month before the warmest day on record in Hobart (41.8 °C on 4 January 2013), the nearby community of Maydena in Tasmania’s southeast experienced the coldest summer day on record (9.4 °C on 4 December 2012) [27]. This variability impedes the ability of the Tasmanian population to adequately acclimatize to heat events over the summer period, potentially increasing vulnerability to heat events when they do occur [32].

While Tasmania has had a state heatwave plan in place since 2013, a paucity of research on heatwaves in Tasmania and their impact on local health systems has hampered efforts by public health policy makers to develop targeted policies and programs to reduce the public health impact of heatwaves. To date, policy and planning has relied on research conducted in other geographic settings, which does not take Tasmania’s unique vulnerabilities or climate into account. 

### 1.2. Research Aim

The aim of this research was to investigate the impact of heatwaves on ED presentations in Tasmania, highlighting similarities and differences with other jurisdictions. Associations with all-cause, age-specific, location-specific, and condition-specific presentations were analyzed.

## 2. Materials and Methods 

### 2.1. Exposure Data

Temperature data from the Bureau of Meteorology Atmospheric high-resolution Regional Reanalysis for Australia (BARRA) dataset [33] were obtained from the Bureau of Meteorology (BoM). BARRA data were used because they provide better spatial and temporal resolution than station data. Averaged maximum and minimum temperatures across a 24-hour period (from midnight to midnight Australian Eastern Standard Time, adjusted from UTC) were used to identify extreme heat events. Heatwaves were identified using the Extreme Heat Factor (EHF) index which was described elsewhere [34]. The index is a relative measure of temperature compared with historical data for each location, and does not rely on meeting an absolute temperature threshold. Using this index, a heatwave is classified as a low intensity, severe or extreme event, where an extreme event is classified as three times the threshold for a severe heatwave event [34]. This method is used by the Australian Bureau of Meteorology for the Heatwave Service for Australia [35] and has been found to be an effective predictor of health service demand during heatwave events [36,37]. Given their impact on health, only severe and extreme events were considered in this analysis. As only a very small number of extreme events were identified, these were combined with severe events for analysis. 

**Figure 1:**
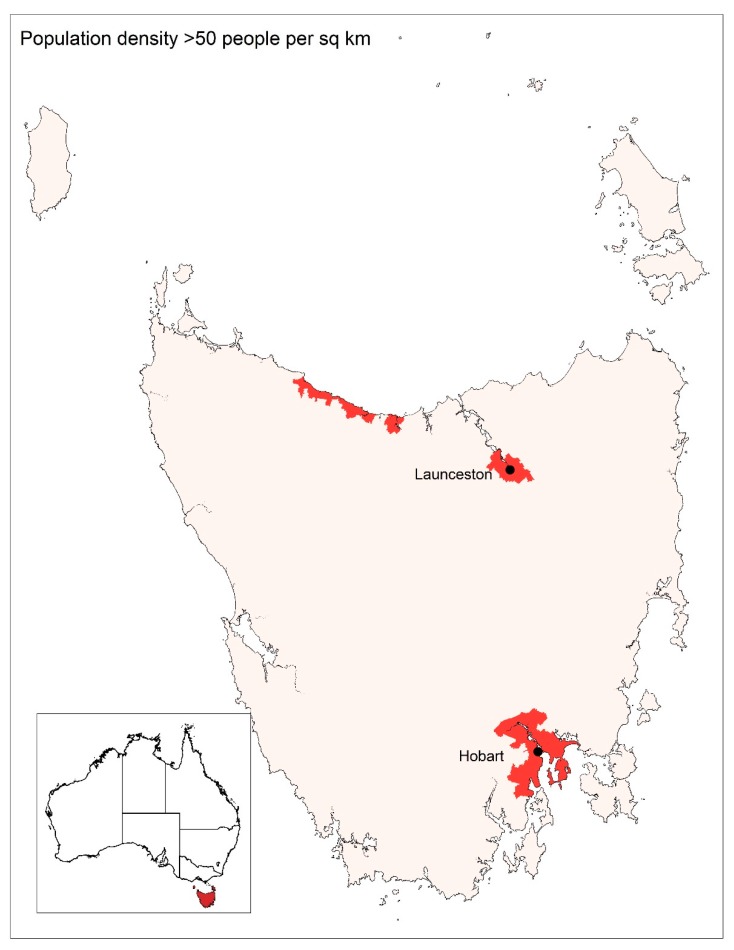
Locations in Tasmania where population density >50 persons per km^2^ [26], inset showing location of Tasmania within Australia.

The BARRA data were matched with the Australian Bureau of Statistics Statistical Area 2 (SA2) regions that displayed a population density >50 persons per km^2^ (see Figure 1). Population density data were sourced from the Australian Bureau of Statistics [25]. 

Air pollution data were obtained from the Environment Protection Authority (EPA) of Tasmania’s air quality monitoring network, known as Base Line Air Network of EPA Tasmania (BLANkET). New Town station data were used to represent Hobart, and Ti Tree Bend station data were used to represent Launceston. Ambient 24-hour (midnight to midnight) average concentrations of particulate matter with a diameter less than 2.5 µm (PM_2.5_) readings were used. Where data-points for a 24–48 hour period were missing, the average of a 7-day period, on either side of the missing data-points were interpolated. Where data-points were missing for longer than 48 hours, data were linearly interpolated using the na.approx() function from the ‘zoo’ package in R [38]. 

State-wide public holidays for Tasmania were obtained using the Python ‘holidays’ package [39]. Locally specific holidays were identified and incorporated.

### 2.2. Outcome Data

ED presentation data were obtained from the Tasmanian Health Service for public hospitals in Tasmania. Only data for Hobart (Royal Hobart Hospital) and Launceston (Launceston General Hospital) were used. This was due to the lack of heatwave events exclusively in the northwest region, compounded with the relatively small number of patient episodes in this much less populated area of the state.

### 2.3. Study Design

This study used a time-stratified case-crossover design. This methodology is commonly used in environmental epidemiology and is suited to a situation where the study population is exposed to a short-term event (e.g., a heatwave), and experiences a health outcome (e.g., an emergency department presentation) [40,41]. Individual presentations, rather than days, are the unit of observation, with each presentation acting as its own control. Environmental data on the date of the health event were compared with that on control days of the same day of the week and within the same calendar month and year.

This methodology has been used previously for similar studies in other locations [42,43] and has been compared to a time-series analysis with analogous results [44,45]. 

The study period was from 1 January 2008 to 31 December 2016.

### 2.4. Analyses

A conditional multivariate logistic regression was performed using the clogit() function from the ‘survival’ package in R [46]. The odds ratio, a measure of the association between an exposure and an outcome [47], and the 95% confidence intervals were calculated for presentations to ED during identified severe/extreme heatwaves. This was performed for the whole population for all conditions combined, and for the following sub-categories:

- Age group (0–5, 0–15 and over 65)
- Gender
- Socio-Economic Index for Areas (SEIFA) category (by suburb of patient address), using the Index of Relative Socio-Economic Disadvantage
- Diagnostic group.

SEIFA categories were amalgamated by condensing scores 1–3 as ‘low advantage’, scores 4–7 as ‘middle advantage’, and scores 8–10 as ‘high advantage’. 

The presenting conditions were classified into diagnostic groups using the International Classification of Disease (ICD-10) codes for the primary diagnosis [48]. Table 1 shows the diagnostic groups and sub-groups analyzed.

The regression model controlled for both observed public holidays and PM_2.5_ for the nearest EPA station.

## 3. Results

In the nine-year period from 1 January 2008 to 31 December 2016, 841,965 people presented to the ED of the Royal Hobart Hospital and the Launceston General Hospital. Characteristics of these presentations are shown in Table 2.

During this period, there were multiple days identified as heatwaves of varying intensities, affecting both regions under study (see Table 3). All identified heatwave days occurred in summer (December to February), where hot days were characterized as arising from hot northerly winds and days of low humidity gave rise to dry heat conditions.

**Figure 2:**
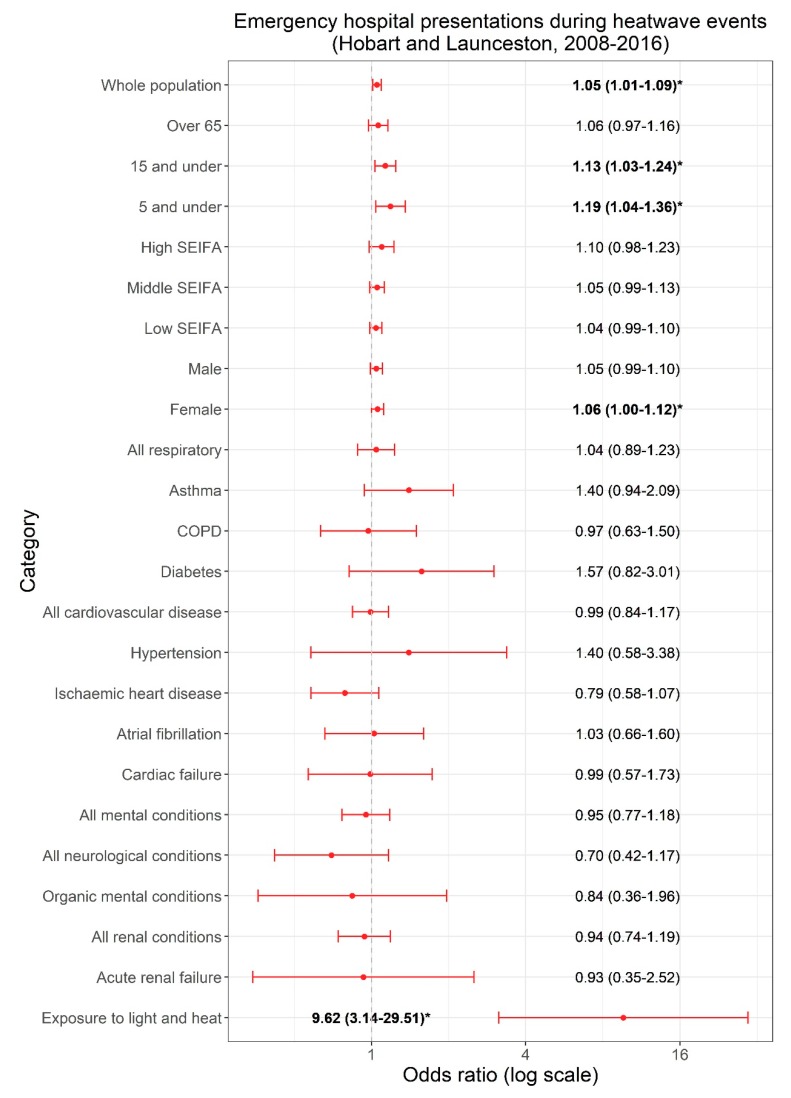
Odds ratios and 95% confidence intervals for the association between ED presentations for specific population characteristics, diagnostic groups, and heatwaves in Hobart and Launceston, Tasmania (2008–2016), adjusted for public holidays and PM_2.5_ (* and bold indicates *p* < 0.05).

Significant associations between ED presentations and identified severe/extreme heatwave days were found (see Figure 2).

ED presentations increased across the whole population (OR 1.05, 95% CI 1.01–1.09), for children aged 15 years and under (OR 1.13, 95% CI 1.03–1.24), and for children aged 5 years and under (OR 1.19, 95% CI 1.04–1.36), while a less precise association in the same direction was found for those aged over 65 years (OR 1.06, 95% CI 0.97–1.16). Results for males and females were similar, although the point estimate was slightly higher in females and attained statistical significance (female OR 1.06; male OR 1.05). There was no clear trend associated with socioeconomic disadvantage. 

A significant association was found for conditions relating to exposure to heat and light (OR 9.62, 95% CI 3.13–29.51). No associations were observed with any other diagnostic subgroups. Results were much less precise due to the smaller number of cases in these subgroups although non-significant elevations in the ORs were observed for asthma (OR 1.40, 95% CI 0.94–2.09), diabetes (OR 1.57, 95% CI 0.82–3.01), hypertension (OR 1.40, 95% CI 0.58–3.38), and atrial fibrillation (OR 1.03, 95% CI 0.63–1.60). Insufficient data were available to perform a conditional logistic regression for psychoses, dementia, and renal calculus, and these conditions were not presented in the results. 

There were no meaningful differences between the crude and adjusted associations (see supplementary data in Table S1 for full results).

## 4. Discussion

In this study, we found that hospital emergency departments in Tasmania’s major population centers experienced a significant increase in presentations (5%) during severe and extreme heatwaves, disproportionately affecting younger age groups. ED presentations increased by 13% for children aged 15 years and under and 19% for children aged 5 years and under. Significant increases in presentations were also found for conditions related to exposure to light and heat (e.g., sunburn and heatstroke). A less precise increase in risk was found for older people, although this group exhibited a similar magnitude to the overall population risk.

Our findings were largely consistent with similar studies in other locations around Australia, showing an association between heatwave events and increases in emergency department presentations [16,49]. Other international studies have demonstrated similar trends in associations between ED presentations and heatwave events [50,51,52]. 

Our findings for increased risk to children in the magnitude observed (2.6x for children aged 15 years and under, and 3.6x for children aged 5 years and under, over the general population) appeared to be unique in the literature. While some studies have demonstrated an elevated morbidity risk to children in heatwaves [53,54], an overwhelming number of studies have consistently highlighted the elderly to be most at risk. This finding warrants further research in the Tasmanian context and has clear policy implications for public health preparedness and communication during heatwave events.

In our study, the difference between ED presentations for males and females during heatwaves was small, showing a slightly higher risk for females. Similar studies, both in Australia and overseas, have demonstrated mixed results for the risk between genders [10,55,56], while some report differences in gender with specific diagnostic conditions [10]. Due to the small number of cases in this study, specific diagnostic conditions were not further analyzed by gender.

Other similar studies have demonstrated that poorer health outcomes appear to be more likely in areas with a greater disadvantage, both in Australia [56,57] and overseas [55,58]. Contrary to expectations, our results did not show a trend in the risk associated with socio-economic disadvantage, however, our ability to identify associations was limited by the lower statistical power in the subgroup analyses. This result also deserved further investigation.

Results of the sub analyses by diagnostic groups were generally less precise due to the smaller numbers of cases evaluated, resulting in wide confidence intervals and no clear associations. Based on similar studies elsewhere, increased risk in cardiovascular, respiratory, renal disease, and mental disease were expected. Recent meta-analyses of cardiovascular and respiratory conditions suggest that mortality is greater than morbidity for these diagnostic groups during heatwaves [59], which might partially contribute to the results found in this study, and deserves further study in the local context.

This study benefitted from analyzing data across a nine-year time frame, indicating ED presentation changes over a number of heatwave events, rather than the analyses of a specific or singular event. Our study also controlled for co-incident air pollution (PM_2.5_) on health outcomes, a well-documented association [60,61,62,63], and for public holidays, which influence the patterns of healthcare utilization.

The results of this study are confined to the relatively small population of Tasmania, making additional sub-categorization analyses difficult to achieve, for example, analyzing the impact of heatwave events on children with asthma [64]. While other similar studies have controlled for ozone [42], these data were not available for the studied population centers and could not be included in this analysis.

While limitations are known to exist with reanalysis data [65], including the possibility of underestimating extremes [66], our study used reanalysis data given the improvement in spatial and temporal resolution offered over observed station data in the study region. Further studies examining the difference between reanalysis and observed data for this region may be warranted but were outside of the scope of our study.

Our findings can assist policy and planning directives in two key areas of health. Detailed planning in Tasmanian hospital emergency departments for heatwave events is now possible, especially as these types of events can be forecast with accuracy in the days prior [67]. This allows for long lead times to accurately adjust rostering and implement surge capacity procedures, potentially minimizing the impact on the hospital system. Secondly, targeted and specific public health preparedness campaigns aimed at the carers of young children, such as parents, child care centers, and schools, can be incorporated into the existing heatwave campaigns and health promotion campaigns already targeting this group, with the aim of reducing the incidence of ED presentations during these events. Neither of these interventions currently exist due to a lack of local evidence.

These findings also allow the issue of self-care in heatwaves to be explored through the media, giving evidence towards heatwaves being a health risk that can be managed. Current media coverage of hot weather tends to focus on recreation opportunities that can be best enjoyed in hot weather [68], rather than emphasizing the potential health issues and mitigation actions.

Further research that analyses the associations between heatwave events and other health care outcomes (for example, mortality, hospital admissions, ambulance dispatches, and GP visits) would assist in strengthening preparedness and response activities, including policy measures associated with extreme heat events in Tasmania.

## 5. Conclusions

This research shows an association between heatwave events and hospital emergency department presentations in the most populated regions of Tasmania, Australia. These associations were apparent across the whole population under study, predominantly for children aged 0–15 and 0–5. These findings may assist in surge-capacity planning for hospital emergency departments during forecast heatwave events, and can help tailor public health preparedness policies for heatwaves. This example of research-to-policy translation highlights the importance of developing well-informed health policy and planning initiatives at a local level, based on local research, demonstrating that while general associations could be made using research from other regions with large-scale studies, specific and targeted responses serve to better inform the local practice. 

## Figures and Tables

**Table 1:** International Classification of Disease (ICD-10) codes for analyzed diagnostic conditions.

Diagnostic Condition	ICD-10 Code
All respiratory	J00–J99
Asthma	J45–J46
Chronic obstructive pulmonary disease (COPD)	J40–J44, J47, J67
Diabetes	E10–E11, E13–E14
All cardiovascular	I00–I99, G45–G46
Hypertensive	I10–I13
Ischemic heart disease	I20–I25
Atrial fibrillation	I48
Cardiac failure	I50
All mental disorders	F00–F99
Dementia	F00–F03
Neuroses	F40–F48
Psychoses	F80–F89
Organic mental disorders (including depression, anxiety)	F00–F09
All renal disorders	N00–N39
Acute renal failure	N17
Renal calculus	N20–N21
Heat and light disorders (including sunburn, heat stroke)	T67, X30

**Table 2:** Characteristics of emergency department (ED) presentations to the Royal Hobart Hospital and Launceston General Hospital for specific population characteristics and diagnostic groups (2008–2016).

Population Characteristic/Diagnostic Group	Total Number (% of Total)	Mean Daily Presentations	Standard Deviation	Minimum/Maximum Presentations
Whole population	841,965 (100%)	256.1	31.4	153/358
Age				
≤5	85,450 (10.1%)	26.0	7.2	5/56
≤15	160,315 (19.0%)	48.8	10.9	18/108
16–65	521,072 (61.9%)	158.5	20.3	90/232
>65	160,500 (19.1%)	48.8	10.3	21/85
Gender				
Male	434,660 (51.6%)	132.2	18.3	80/201
Female	407,032 (48.3%)	123.8	17.6	67/181
SEIFA				
Low	437,577 (52.0%)	133.1	17.6	75/194
Middle	252,039 (30.0%)	76.7	11.7	36/118
High	135,392 (16.0%)	41.2	8.7	15/78
All respiratory	67,439 (8.0%)	20.5	7.6	3/63
Asthma	8546 (1.0%)	2.7	1.6	1/10
COPD	10,365 (1.2%)	3.4	1.9	1/14
All cardiovascular	49,436 (5.9%)	15.0	4.3	3/31
Cardiac failure	5199 (0.6%)	2.0	1.1	1/9
Hypertensive	1312 (0.2%)	1.2	0.5	1/5
Atrial fibrillation	2724 (0.3%)	2.2	1.2	1/8
Ischemic heart disease	13,964 (1.7%)	4.3	2.1	1/15
Diabetes	1994 (0.2%)	1.3	0.6	1/5
All mental disorders	34,509 (4.1%)	10.5	3.7	1/27
Dementia	655 (0.1%)	1.3	0.4	1/4
Neuroses	6459 (0.8%)	2.3	1.3	1/9
Organic mental	2639 (0.3%)	1.5	0.8	1/7
Psychoses	21 (0.002%)	1.0	0	1/1
All renal	20,914 (2.5%)	6.4	2.6	1/19
Acute renal failure	1416 (0.2%)	1.3	0.5	1/5
Renal calculus	120 (0.01%)	1.1	0.3	1/2
Exposure to light and heat	199 (0.02%)	1.3	1.1	1/12

**Table 3:** Number of days identified as heatwave days for each region, at each heatwave intensity.

Region	Low Intensity Days	Severe Days	Extreme Days
South (Hobart)	85	9	1
North (Launceston)	153	18	5

## Supplementary Materials

The following are available online at https://www.mdpi.com/1660-4601/16/19/3715/s1, Table S1: Crude and adjusted results.
